# Microwave versus radiofrequency ablation for the treatment of liver malignancies: a randomized controlled phase 2 trial

**DOI:** 10.1038/s41598-021-03802-x

**Published:** 2022-01-10

**Authors:** Aleksandar Radosevic, Rita Quesada, Clara Serlavos, Juan Sánchez, Ander Zugazaga, Ana Sierra, Susana Coll, Marcos Busto, Guadalupe Aguilar, Daniel Flores, Javier Arce, José María Maiques, Montserrat Garcia-Retortillo, José Antonio Carrion, Laura Visa, María Villamonte, Eva Pueyo, Enrique Berjano, Macarena Trujillo, Patricia Sánchez-Velázquez, Luís Grande, Fernando Burdio

**Affiliations:** 1 Department of Radiology, Hospital del Mar de Barcelona, Passeig Maritim 25-29, 08003 Barcelona, Spain; 2 Department of Experimental and Health Sciences, Universitat Pompeu Fabra, Barcelona, Spain; 3 Hepatology Section, Gastroenterology Department, Hospital del Mar. IMIM, Barcelona, Spain; 4 Department of Oncology-IMIM-Ciberonc, Hospital del Mar, Barcelona, Spain; 5 Department of Surgery, Hospital del Mar, Barcelona, Spain; 6 BioMIT, Department of Electronic Engineering, Universitat Politècnica de València, Valencia, Spain; 7 BioMIT, Department of Applied Mathematics, Universitat Politècnica de València, Valencia, Spain

**Keywords:** Cancer therapy, Clinical trials, Randomized controlled trials

## Abstract

Microwave (MWA) and radiofrequency ablation (RFA) are main ablative techniques for hepatocellular carcinoma (HCC) and colorectal liver metastasis (MT). This randomized phase 2 clinical trial compares the effectiveness of MWA and RFA as well as morphology of corresponding ablation zones. HCC and MT patients with 1.5–4 cm tumors, suitable for ablation, were randomized into MWA or RFA Groups. The primary endpoint was short-to-long diameter ratio of ablation zone (SLR). Primary technical success (TS) and a cumulative local tumor progression (LTP) after a median 2-year follow-up were compared. Between June 2015 and April 2020, 82 patients were randomly assigned (41 patients per group). For the per-protocol analysis, five patients were excluded. MWA created larger ablation zones than RFA (*p* = 0.036) although without differences in SLR (0.5 for both groups, *p* = 0.229). The TS was achieved in 98% (46/47) and 90% (45/50) (*p* = 0.108), and LTP was observed in 21% (10/47) vs. 12% (6/50) (OR 1.9 [95% CI 0.66–5.3], *p* = 0.238) of tumors in MWA vs. RFA Group, respectively. Major complications were found in 5 cases (11%) vs. 2 cases (4%), without statistical significance. MWA and RFA show similar SLR, effectiveness and safety in liver tumors between 1.5 and 4 cm.

## Introduction

Thermal ablation is the first-line option for patients with small (< 2 cm) hepatocellular carcinoma (HCC), who are not potential liver transplant candidates, and in patients with up to three 3 cm or smaller nodules with associated diseases, according to the Barcelona Clinic Liver Cancer group (BCLC)^1^. On an individual basis, ablation can also be considered in patients with larger tumors (3–5 cm) and advanced liver disease (Child–Pugh score B)^1,2^. Likewise, the European Society for Medical Oncology (ESMO) also includes thermal ablation as part of the treatment algorithm for oligometastatic colorectal disease^3^. Most authors agree that ablation is considered for small (< 3 cm) colorectal liver metastasis (MT) in patients who are unsuitable for resection due to impaired general health, a history of extensive abdominal surgery, lesions in an unfavorable location or insufficient future liver remnant^4^.

Radiofrequency ablation (RFA) is a mature and well-known thermal ablation technique which has been shown to be both safe and effective for treating liver nodules^2,5,6^. However, liver tumors adjacent to large vessels may often be incompletely ablated due to the heat sink effect, which can lead to *Local Tumor Progression* (LTP)^5,7^. Microwave ablation (MWA) using the most advanced devices obtains shorter ablation times, higher ablation temperatures, larger ablation zones and a weakened heat-sink effect than RFA, all of which are considered to improve the efficacy of tumor ablation^8–11^. However, these theoretical advantages of MWA could also be its flaws because being more effective it can easily injure adjacent critical structures (e.g. nearby bile or portal structures)^12^. This is mainly because MWA power can propagate thorough materials with much lower electrical conductivity than RFA. RFA requires an electrically conductive path, which is interrupted when the electrodes enter desiccated tissue, which inevitably appears after a certain application time^10,11,13,14^. The actual ablation zone created by MWA may thus be larger with grater tissue contraction, but not readily apparent on immediate post-ablation imaging^15^ and may be more elongated or more ellipsoid than RFA (i.e. with a lower *Short to Long diameter Ratio* -SLR-)^16–19^. Within these ellipsoid ablation zones, an additional area of healthy tissue is needlessly ablated and this could result in larger ablation volumes and potentially higher rate of complications. For many authors the major limitation of conventional MWA systems is the fact of being unable to predict ablation zone size and shape^5,20^. However, ablation zone predictability is expected to improve with advanced MWA systems^5,20,21^.

Several recent randomized clinical trials (RCT) have compared different classical RFA devices (e.g. cool-tip applicators) with the latest MWA devices used to treat a combination of multiple small and medium-sized tumors^21–25^. These RCT often obtained similar or slightly better LTP for MWA, insufficient in order to draw definitive conclusions. For example, Vietti Violi et al.^24^ observed 6% and 12% LTP at 2 years for MWA and RFA, respectively, even though these differences did not reach statistical significance (median follow-up 26 months). Only one RCT^24^ reported the volume of ablation zones. No other parameters of ablation zone morphology have been reported in RCTs so far. The aim of the present RCT was therefore not only to compare the effectiveness of MWA and RFA in liver tumors (1.5–4 cm diameter) in term of TS and LTP rate, but to compare the morphology of ablation zones created with these devices. The MWA procedure was performed by means of an up-to-date technique (2.45 GHz generator) and the RFA by means of a hybrid applicator (cool-tip applicator with hyperosmotic saline infusion) previously shown to create larger and more spherical ablation zone in comparison with conventional cool-tip applicator^26–28^.

## Material and methods

### Study design

A prospective, randomized, parallel-group, single-blinded phase 2 clinical trial was conducted by the Department of Radiology and Surgery at the Hospital del Mar, a tertiary care hospital in Barcelona, Spain. The study protocol was approved by the Clinical Research and Ethics Committee of the Hospital (June 27th, 2012, Ref 2012/4776/I) and by the Spanish Agency of Medicines and Medical Devices (June 10th, 2013, Ref: 438/13/EC). All patients gave their written informed consent before the procedure. The study was conducted in accordance with principles of the Declaration of Helsinki and Good Clinical Practice guidelines and was officially registered under ISRCTN73194360 on 13/04/2015 on https://www.isrctn.com providing additional study information.

### Study population

Between June 2015 and April 2020, all the HCC or MT patients suitable for local ablation were assessed for eligibility after being discussed at the multidisciplinary tumor board for liver malignancies. Inclusion criteria were: age 18 years or older, a diagnosis of HCC or MT suitable for ablation assessed by cross-sectional imaging or biopsy (according to BCLC classification^1^ or ESMO guidelines^3^), tumor number at presentation ≤ 3 and the largest tumor diameter between 1.5 and 4 cm. Both percutaneous and surgical approaches were acceptable. The exclusion criteria were: American Anesthesia Association (ASA) Classification > III, cardiac pacemaker, thrombocytopenia < 50,000/ mL and previous biliodigestive anastomosis.

### Randomization, concealment and masking

The patients who agreed to participate were randomly assigned to either the MWA or RFA Group. Block Randomization^29^ (1:1) was used to reduce bias and achieve a balance in the allocation of participants to treatment arms without taking into account any other variable in the randomization process. Patient allocation and assignment was done by a non-clinician co-author (RQ) not involved in the care of the patients^30^. The patients were masked for the treatment but not the physicians, since different devices were used.

### Procedures

A pre-interventional multiphase abdominal CT or MRI scan no older than 30 days was performed and same standard radiological protocol was used for all patients (basal, arterial, venous and delayed phase scan with 2.5–3 mm slice thickness). All nodules were carefully assessed and recorded for size, segment distribution and unfavorable tumor locations. Unfavorable locations were defined as those 5 mm or closer to critical structures, including the gallbladder, gastrointestinal tract, liver hilum, pericardium, diaphragm, and major vessels (larger than 3 mm)^31^.

All the liver tumors included and the targeted ablation zones were semi-automatically delineated on consecutive axial images using Advantage Window Workstation Volume Viewer software (GE Healthcare, Milwaukee, WI, USA) and their volumes were calculated on the portal venous phase acquisition with 2-mm slice reconstruction at the preprocedural and postprocedural (1-month after) CT (Figs. S1, S2), as recommended by Ahmed et al.^32^. MRI was used for ablation zone calculation in only two patients (with three tumors), due to low conspicuity of the initial tumors at CT images. The digital images were saved and processed on 3D Doctor software (AbleSoftware, Lexington, MA, USA), which extracts information from medical image files to create 3D models. The axial and the two corresponding transverse diameters were delineated manually (using multiplanar reconstruction and 3D volume rendering similarly to Heering et al.^33^). All the measurements were performed by the same radiologist (AR).

Three radiologists (AR, AZ and JS) with 5–10 years of experience in interventional oncology performed the ablation procedures. A single session complete tumor ablation with a 5 mm ablation margin was the overall target. However, in cases of subcapsular and juxta-vascular locations these margins were not achievable. For tumors up to 2.5 cm, distant from sensitive structures, large vessels or diaphragm, we performed single ablation. In all other circumstances, an overlapping ablation were performed and the number of ablations directly depended on the tumor size, proximity to sensitive structures and to the diaphragm, tumor visibility, desirable applicator placement as well as ablation progression during the procedure. The ablation was performed during open and laparoscopic surgery in two patients. In two additional patients, percutaneous ablation of deep tumors was performed in an operating room, followed by laparoscopic resection of distinct, synchronic tumors. In the rest of the patients, ablation was performed percutaneously under conscious sedation with ultrasound guidance in a dedicated ultrasound suite. An artificial ascites or artificial pleural effusion was created in selected cases of subcapsular tumors in order to protect the surrounding structures or to improve visualization of the tumor or to be used as path for applicator insertion. Ascites was created following the technique described by Rhim^34^. We used our own modification of this technique for pleural effusion creation: (1) patients were instructed to completely exhale and hold their breath, (2) an 18-gauge sheathed needle was slightly (0.5–1 cm) introduced into the subphrenic portion of liver parenchyma just below the edge of the lung, orthogonal to the skin surface, (3) the inner stylet of the sheath needle was removed, a 0.035-inch guidewire inserted into the sheath and the patients instructed to fully inhale and hold their breath, (4) the guidewire was pushed into the pleura and the 6-Fr catheter was introduced over the guidewire.

MWA was performed using a 2.45 GHz MW ablation generator (HS AMICA, HS Hospital Service, Rome, Italy) with a maximum output of 140 W and a cooled mini-choked 14-gauge antenna. A 3–6 min application at 60–80 W was standard, according to the manufacturer’s instructions, as in Amabile et al.^35^. RFA was conducted with a 200 W generator (CC-3, Radionics, Burlington, MA, USA). RF energy was delivered in impedance control mode with a 1-min ramp-up phase followed by 3–5 min at maximal power. A single 14-gauge, 3-cm long internally cooled electrode with two electrically isolated expandable needles to infuse hypertonic (20%) saline at 2 mm from the electrode surface (Gnomon, Apeiron Medical, Valencia, Spain) was used following the recommendations described elsewhere for both animals^26,28^ and patients^27^. Two 0.5 ml boluses were injected at 0 s and 90 s which gives 2 ml total volume injected per ablation. At the end of the procedure, the ablation zone was roughly assessed by non-enhanced ultrasound. At the discretion of the operator an immediate contrast-enhanced CT control scan would be performed and if considered the margins would be enlarged during the same session. The active ablation time and number of applications of the ablated tumors were recorded.

Radiological assessment of the ablation procedure was performed 1 month after the intervention, then every 3 months during the first two years and finally every 6 months. In the case of any residual unablated tumor, an additional ablation procedure was performed using the same technique. *Primary Technical Success* (TS) and LTP were defined as tumor with 5 mm-safety margin being completely covered by the ablation zone (at 1-month follow-up) and the appearance of any tumor focus in contact with the ablation zone, respectively, as defined by Ahmed et al.^32^. *Distant hepatic progression* was defined as any new hepatic lesion not in contact with the ablation zone. New extrahepatic lesions were considered as metastatic disease. The date of the recurrent disease was determined by the first imaging study showing unequivocal recurrence to ascertain *Time to Tumor Progression* (TTP).

### Outcomes

The primary endpoint was SLR^19^ assessment and the secondary endpoints were TS (at the one-month control scan) and the cumulative incidence of LTP after 2 years of follow-up, The main study outcomes were also sphericity ratio (SR), surface area of the ablation zone^36^ and the coefficient of variation (CV)^26,37^ as a measure of reproducibility. SR referred to ratio of axial diameter to the average of the two transverse diameters of the ablation zone and closer to 1 implies greater sphericity^37^. The secondary endpoints were safety and overall survival (OS)^32^. Safety represented all treatment-related complications classified according to the Society of Interventional Radiology guidelines by treated tumor basis taking into account the highest grade^38^.

### Sample size and statistical analysis

Given that not only the expected size but the shape of ablation zone is essential for successful tumor ablation^39^, the sample size was based on morphological parameter—SLR as the main variable. Based on the historic difference in SLR^19,26^ with similar methods of ablation (MWA-Amica (MWA Group) < Cool Tip and Cool-Tip < Gnomon (RFA Group), it was estimated that SLR in the MWA Group would be reduced by 15% compared to RFA Group. To achieve a power of 80% in detecting differences with a 2-sided test and a type I error of 0.05, a total of 82 tumors were estimated to be required. All the analyses were performed on the per-protocol population. Since the patients could have multiple lesions, we expected the minimal number of lesions to be greater than the number of patients. Differences in the quantitative data distributions between the two groups were assessed by a two-tailed unpaired Student’s *t* test. Differences in frequencies for categorical data and the proportion of lesions with TS and cumulative incidence of LTP after 2-year follow-up were assessed by the two-tailed χ^2^ test. The Odds-Ratio (OR) was calculated with 95% Confidence Intervals (CI). OS, TTP and LTP were assessed through Kaplan Meier curves using the Log-rank test. Univariate regression with Cox analysis was also calculated to compare time to progression between the two groups. A p-value less than 0.05 was considered to be significant.

## Results

**Figure 1:**
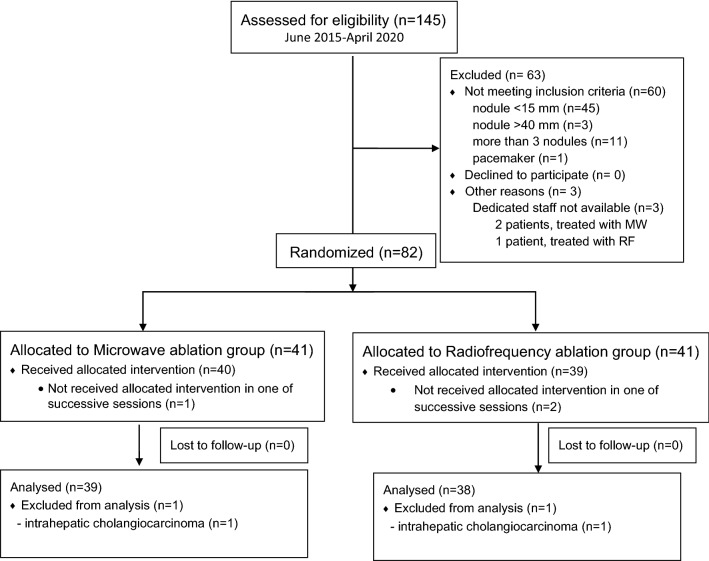
Clinical trial profile.

Between June 9, 2015 and April 30, 2020, 145 patients were considered for the trial and 63 (44%) were excluded for not meeting the inclusion criteria or other reasons (Fig. 1). The remaining 82 patients were randomized to MWA Group (n = 41) or RFA Group (n = 41). Three patients (4%) partially received the allocated intervention and were excluded. Two of these patients received allocated intervention (RFA) for treatment of HCC during the initial session, but did not receive the same intervention for metachronous tumor in further session due to procedural errors. The third patient, with bifocal HCC, received allocated intervention (MWA) of one tumor, as planned, but the ablation of the second tumor was not carried out. Two (2%) additional patients were excluded from the analysis due to other diagnoses (intrahepatic cholangiocarcinoma). We considered that a single arterial-phase enhancing tumors in both patients were likely to be HCC and we randomized the patients. However, due to absence of late wash out, the biopsy was performed just prior the ablation during the same session. The final diagnosis of intrahepatic cholangiocarcinoma was established and both patients were excluded. LTP was not observed in any of these treated tumors. The final study population was thus 77 patients with 136 tumors (97 larger than 1.5 cm) (Fig. 1 and Table 1). As shown in Table 1, patient demographics, diagnosis, ablated tumors per patient, HCC risk factors among others did not differ between the groups, except for a higher age in MWA Group (mean value: 75 vs. 67 years, *p* = 0.003).

**Table 1:** Patient data.

	MWA (n = 39)	RFA (n = 38)	*P*
Age at inclusion, years	75 (46–93)	67 (48–84)	0.003
Men	22 (56%)	29 (76%)	0.065
**Diagnosis**			0.742
Hepatocarcinoma	30 (77%)	28 (74%)	
Liver metastases	9 (23%)	10 (26%)	
Number of Ablated Tumors*	67	69	0.661
Tumors ≥ 1.5 cm	47	50	0.381
Tumors ≥ 3 cm	12	12	0.861
Number of tumors ablated per patient*			0.468
**1**	22 (56%)	22 (58%)	
**2**	9 (23%)	8 (21%)	
**3**	7 (18%)	4 (11%)	
> 3	1 (3%)	4 (11%)	
Cirrhosis	77%	71%	0.549
**BCLC stage**			0.945
0	2 (5%)	2 (5%)	
A	28 (72%)	26 (69%)	
N/A	9 (23%)	10 (26%)	
**Child–Pugh score**			0.257
A	28 (72%)	21 (55%)	
B	2 (5%)	6 (16%)	
N/A	9 (23%)	11 (29%)	
Patients with previous local treatments**	10 (26%)	8 (21%)	0.634
Concomitant liver surgery	3 (8%)	1 (3%)	0.615

Data are n (patients), n (%), or the mean (range).

*Include all liver tumors ablated during the trial (tumors < 1.5 cm were not considered for analysis).

**Include previous liver surgeries or liver ablations; previously treated tumors were not considered for the trial. Analyses are done by patient.

There were no differences between the tumor characteristics of both groups, particularly in tumor size and volume (Table 2). All ablation zones were evaluated through echography during the procedure, while a total of 29 tumors (62%) in the MWA Group and 33 tumors (66%) in the RFA Group (*p* = 0.680) were further evaluated by immediate post-ablation CT control scan. A total of 13 tumors (45% of those tumors, 27% of total number of ablated tumors) in the MWA Group and 12 tumors (36% of those tumors, 24% of total number of ablated tumors) in the RFA Group were re-ablated during the same session in order to ensure sufficient margins (*p* = 0.680). With similar number of total ablations per tumor between both groups, the ablation zones were larger in MWA than in RFA Group (36.8 cm^3^ vs. 27.7 cm^3^, *p* = 0.036). The shape of the ablation zone was slightly different in the long diameter (*p* = 0.01) but with similar SLR, SR and CV. The surface area seemed to be larger and more variable in MWA Group but it did not reach statistical significance (Table 2). Overlapping ablation (67/97 (69%) of tumors) created larger ablation zones than single ablation (35.5 cm^3^ vs 24.7 cm^3^, *p* = 0.020) although without differences in sphericity ratio (1.73 vs 1.68, *p* = 0.523) or SLR (0.55 vs 0.52, *p* = 0.328). At the 1-month CT, one (2%) of 47 tumors in MWA Group and five (10%) of 50 tumors in RFA Group had signs of residual tumor (*p* = 0.108). All these tumors were treated again with the same technique and all achieved complete response after this additional procedure. In MWA Group we included MT (11 tumors) and HCC (36 tumors) and no differences were found between sub-groups regarding tumor size (2.7 vs. 2.4 cm, *p* = 0.145), ablation zone volume (44.6 vs. 33.8 cm^3^, *p* = 0.122), long diameter of ablation zone (6.4 vs. 5.6 cm, *p* = 0.760), SLR (0.48 vs. 0.52, *p* = 0.956), TS (91% vs. 100%, *p* = 0.234), LTP (28 vs. 19%, *p* = 0.234) and TTP (22.5 vs. 18.5 months, *p* = 0.401). Nor were the differences found in RFA Group including 13 MT and 37 HCC tumors: tumor size (2.3 vs. 2.4 cm, *p* = 0.571), ablation zone volume (29.8 vs. 27.1 cm^3^, *p* = 0.644), long diameter of ablation zone (5.2 vs. 5.2 cm, *p* = 0.943), SLR (0.58 vs. 0.53, *p* = 0.340), TS (100% vs. 86%, *p* = 0.309), LTP (0 vs. 16%, *p* = 0.168) and TTP (27 vs. 19 months, *p* = 0.100). No differences in technical success (55/59 (93%) vs. 36/38 (95%)) or LTP (10/59 (17%) vs 6/38 (16%) of nodules, *p* = 0.880) were observed in subgroup of patients with vs. without artificial ascites/pleural effusion. All patients with colorectal carcinoma were initially operated on the primary site.

**Table 2:** Treated tumor information.

	MWA group (n = 47)	RFA group (n = 50)	*p*-value
Tumor Size (cm)	2.5 (1.5–4.0)	2.4 (1.5–4.0)	0.490
Tumor volume (cm^3^)	5.6 (0.6–26.8)	6.3 (0.3–28.5)	0.619
Number of ablations per tumor	2.3 (1–6)	2.6 (1–7)	0.313
Ablation time (min) per tumor	8.8 (2–25)	11.2 (4–32)	0.068
Nodules treated with consecutive overlapping ablations	30 (64%)	37 (74%)	0.279
Deposited energy (kJ) per tumor	40.4 (9.6–120.0)	92 (27.1–288.0)	–
**Size and shape of ablation zone**			
Long diameter (cm)	5.8 (3.5–9.2)	5.2 (3.5–8.2)	0.01
Short diameter (cm)	2.9 (1.7–5.2)	2.8 (1.2–5.2)	0.282
Short to long diameter ratio -SLR-	0.5 (0.3–0.7)	0.5 (0.3–0.9)	0.229
Volume (cm^3^)	36.8 (9.6–98.3)	27.7 (4.9–101)	0.036
Ablation to tumor volume ratio-ATR-	11.4 (2.5–95.0)	10.3 (1.1–72.7)	0.672
Sphericity ratio	1.7 (1.2–3.1)	1.7 (1.1–2.8)	0.490
Coefficient of variability	65%	64%	-
Surface area (cm^2^)	70.6 (14.9–151.0)	62.5 (22.9–139.6)	0.173
Unfavorable location*	34 (72%)	37 (74%)	0.854
Artificial ascites/pleural fluid	29 (62%)	30 (60%)	0.864
Technical Success (TS)	46 (98%)	45 (90%)	0.108
Cumulative incidence of local tumor progression after mean 2-year follow-up (LTP)	10 (21%)	6 (12%)	0.234

Data are n (number of tumors), n (%), or the mean (range). All data refer to tumors > 1.5 cm (see inclusion criteria).

*See text for definition. Ablations of local recurrences are not included. Analyses are by tumor.

Postprocedural complications are shown in Table 3. Thirteen (27%) ablated tumors in the MWA and 11 (22%) in the RFA Group led to complications: two grade 1, fifteen grade 2, four grade 3 and three grade 4 complications were reported, with no significant differences between the groups (*p* = 0.519). No treatment-related deaths were reported.

**Table 3:** Complications.

	MWA group (n = 47)	RFA group (n = 50)
**Grade 1**	0 (0%)	2 (4%)
Asymptomatic biloma	0	1
Peripheral portal vein branch thrombosis	0	1
**Grade 2**	8 (17%)	7 (14%)
Pain requiring additional medication	4	3
Abdominal wall burns	0	1
Postablative syndrome	4	3
**Grade 3**	2 (4%)	2 (4%)
Transitional encephalopathy	0	1
Delayed bilio-bronchial fistula	1	0
Abscess	1	1
**Grade 4**	3 (6%)	0 (0%)
Delayed biliar fistula	1	0
Abscess	1	0
Acute cholecystitis	1	0
**Grade 5**	0	0

Data are n, n (%). Analyses are by ablated tumor.

**Figure 2:**
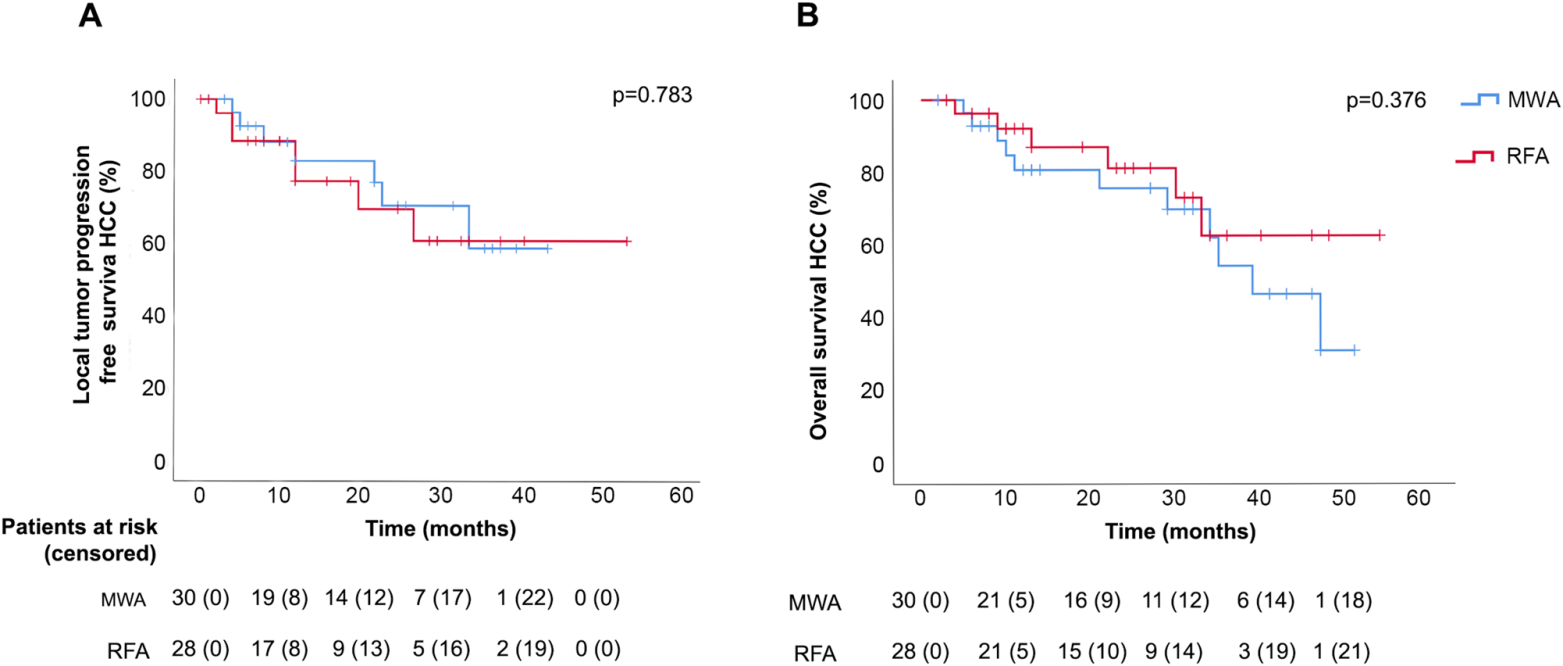
Kaplan–Meier curve of local tumor progression free survival (**A**) and overall survival (**B**) in HCC by group (MWA: microwave ablation; RFA: radiofrequency ablation).

The median follow-up was similar in both groups (27 months [IQR 19–28] in MWA Group vs. 23 months [IQR 18–28] in RFA Group; *p* = 0.861). After mean 2 years follow-up, the cumulative incidence of tumors with LTP did not differ between groups (10 of 47 tumors in MWA Group vs. 6 of 50 in RFA Group, OR = 1.9 [95% CI 0.66–5.3], *p* = 0.238). Neither did the Kaplan–Meier analysis of LTP by patient differ between the groups, either in HCC (see Fig. 2A) or in liver metastases (*p* = 0.071).

Median TTP was also similar between the MWA and RFA Groups in both diagnoses, in particular 27 months [95% CI 19–36] vs. 19 months [95% CI 1–36] (*p* = 0.329), respectively, hazard ratio 1.4 [95% CI 0.6–3.1] (*p* = 0.413), for HCC and 17 months [95% CI 9–25] vs. 6 months [95% CI 4–8] (*p* = 0.413), respectively, hazard ratio 1.1 [95% CI 0.3–3.6] (*p* = 0.925), for MT. There were no differences in overall survival between groups in both diagnoses. The 2-year survival rate was 76 vs. 81%, respectively, hazard ratio 1.6 [95% CI 0.6–4.3] (*p* = 0.378), for HCC (see also Kaplan–Meier analysis in Fig. 2B) and 88% vs. 56%, respectively, hazard ratio 0.5 [95% CI 0.9–2.8] (*p* = 0.440), for liver metastasis.

## Discussion

The main finding of this randomized clinical trial is that hyperosmotic saline enhanced radiofrequency ablation for treatment of hepatocellular carcinoma and colorectal liver metastasis (tumor size 1.5–4 cm) created ablation zones with similar short to long diameter ratio as microwave ablation. The long diameters of ablation zones were shorter and the corresponding volumes were smaller in radiofrequency group, although without difference in short diameter, sphericity ratio or oncological outcomes in either group. No difference between two groups in rate of primary technical success, time of ablation, cumulative incidence of local tumor progression, time to progression, overall survival or rate of complications during mean 2-year follow-up was observed.

Local tumor progression in tumors under 2 cm, even with classical RFA devices, is less than 1%^40,41^ and in liver tumors over 4 or 5 cm may lead to an unacceptable rate of local tumor progression, sometimes over 60% in classical references^40,42^. In any case, the selected tumor size group (1.5–4 cm in diameter) is the everyday challenge of any physician performing thermal ablations, which is why we focused our study on this tumor size group. Several retrospective^6,31,43,44^ and randomized clinical trials^19,22,24,45^ compared microwave and radiofrequency ablation systems dealing with primary or secondary tumors or both. Some of these, however, were carried out with first line thermoablative systems, as in the RCT by Shibata et al.^45^, which treated patients with HCC nodules smaller than 4 cm in diameter and found equivalent results between both techniques with a 24% cumulative incidence of local tumor progression in the MWA Group. The RCT conducted by Abdelaziz et al.^22^ could have been limited by its short follow-up (not specified) and a high lost to follow-up (52%) ^46^. The RCT conducted by Vietti Violi et al.^24^ compared MWA and RFA in treating HCC in up to three lesions of 4 cm or less should be highlighted for its well established design. In this last study the authors observed 6% and 12% LTP at 2 years for MWA and RFA with no significant differences between groups (median follow-up of 26 months). However, the lesion size in this study was smaller than in our study (1.8 cm vs. 2.4 cm) due to the different inclusion criteria. In the present study, after excluding tumors smaller than 1.5 cm, and with a mean follow-up of 25 months (complete duration of the study: 58 months and no patients lost to follow-up), we neither observed differences in cumulative incidence of LTP between groups (21% and 12%, *p* = 0.234 for MWA and RFA, respectively). It also gave an insight into the long-term outcomes not only in HCC but also in MT with an inherently different natural history.

MWA was initially considered to be less safe than RFA because the ablation zone shape was considered less predictable^17,47,48^. For example, Van Tilborg et al.^47^, in a retrospective study with 774 colorectal metastases treated by MWA or RFA in 243 patients, observed that biliary complications (biloma/biliary leakage, biliary obstruction and bilio-pleural fistula) were especially common after peribiliary-MWA (57%) vs. peribiliary-RFA (3%) and these significant differences did not decrease with operator experience. However, in a multicenter Italian study based on 1037 MWA in 736 patients, major complications were found in only 2.9% of the patients -similar to historical RFA procedures^20^. Nor were there any significant differences in device-related complications found in any of the available RCTs comparing MWA and RFA^19,22,24,45^. In our study major complications (grade 3–4) were found in 5 cases in the MWA Group (11%) vs. 2 cases in the RFA Group (4%). Although there was a trend toward higher rate of major complications in MWA Group, the difference was not significant. It is noteworthy that the case of cholecystitis was not directly caused by ablation and that broncho-biliar and hepato-biliar fistulas developed as late complications after infection of previously untreated bilomas (6 months after the ablations in both cases).

MWA and RFA destroy tissue via thermally coagulative necrosis. However, because of their inherently different energy deposition mechanisms many differences in terms of ablation zones can be seen^10,14^. Many improvements have been seen in recent years in thermal deposition during ablation, particularly in MWA, which makes it difficult to compare different devices based on the same technology^14^. Even with the latest generation of devices, ablation zone shapes created by MWA tend to be larger, more elongated and maybe less affected by the heat-sink effect^10,16–18,49^, but usually have less control of heat propagation than RFA, possibly because MWA is highly dependent on tissue properties (water content)^10,14,15^. In our study, after a careful 3D reconstruction of each ablation zone, we confirmed that MWA created larger ablation zones mainly because of their more elongated shape (although without significant impact on SLR) but were not significantly less predictable taking sphericity ratio, coefficient of variability and surface area into account. SLR as the sole criterion may not be the best indicator of ablation effectiveness. However, together with other parameters of ablation zone morphometry, in particular with the short diameter of ablation zone (identical for two systems used in this trial) it allows the comparison of the ablation zone geometry to be more reliable. Although we expected to find, we did not find differences in SLR between RFA and MWA group included in this randomized clinical trial. It could be partially attributed to the differences in tumor size, tumor type, overlapping ablation and tumor locations when compared with the previously published studies^19,26^.

A high number of tumors at unfavorable location has been included in this trial (72% in MWA Group and 74% in RFA Group). We did not find differences in term of primary technical success (26/26 (100%) vs. 65/71 (92%) of tumors, *p* = 0.187) or cumulative local tumor progression during mean 2-year follow-up (5/26 (19%) vs. 11/71 (15%) tumors, *p* = 0.488) for tumors at favorable vs. unfavorable locations. Contrast enhances ultrasound or immediate CT/RM contrast enhanced scan could be helpful in such cases in order to ensure complete ablation. We consider that the most challenging ablations are those of hilar tumors and of subcapsular tumors at the dome of segments 4 and 8. Ascites not only protects the surrounding structures but we consider it also reduce the adhesions formation enabling the ascites formation for future procedures. It also reduces respiration dependent liver movements thus reducing inadvertent applicator displacements. Artificial pleural effusion could be useful in selected cases of hepatic dome or high posterior right liver lobe tumors when creation of artificial ascites was not feasible (due to postsurgical adhesions) or when desirable effects of ascites were not achieved or when a transpleural approach was mandatory for the proper applicator orientation. Depending on center expertise, laparoscopic approach could be considered in some of these cases. Probably, for liver tumors next to central bile ducts and large vessels, irreversible electroporation or chemo-electroporation could be an option.

This study had several limitations: first, the trial was performed in a single third referral center with a special dedication to these procedures and experienced in treating tumors at unfavorable locations. Although our results are in line with previous RCT results, it is conceivable that some results may differ with different patient inclusion criteria. We included both HCC and liver MT. However, in subgroup analysis, there was no difference regarding tumor size, ablation zone volume, long diameter of ablation zone, SLR, TS, LTP and TTP between HCC and MT neither in MWA Group nor in RFA Group. Second, to reduce the risk of device-related bias we chose to use only one advanced needle-shaped device in each group, even though there are numerous commercially available devices with similar characteristics. In any case, we have to acknowledge that accurate comparisons between MWA and RFA systems are difficult. Third, as in other RCT in this field^21,24^, per protocol analysis was performed to correctly evaluate the true efficacy of each technology even though this could be deemed a sub-optimal approach in general terms. Five patients were excluded from analysis. Fourth, the sample size of the study was based on ablation zone morphology (SLR) and not on other variables like TS or LTP which may be more reliable especially when overlapping ablations are performed. However, TS or LTP are difficult to estimate in advance for new methods of ablation. Fifth, although a single ablation per tumor is preferable to achieve a reproducible evaluation of ablation zone shape, we performed a mean of more than two ablations per tumor in a pragmatic approach. No differences were found between both groups regarding number of ablations per tumor and proportion of tumors with overlapping ablations. Although overlapping ablations zone volumes were larger, no differences were observed between overlapping and single ablation zones regarding SLR and sphericity ratio. Nevertheless, overlapping ablations could mask differences in ablation zone shape.

## Conclusion

This randomized study showed that MWA created larger ablation zones than RFA although with similar ablation zone shapes (expressed through short to large diameter ratio) and with similar technical success and local tumor progression rate in liver tumors between 1.5 and 4 cm. After a mean 2-year follow-up, we observed no significant difference in the rate of complications, median time to progression or overall survival, considering that both modalities could be a good treatment option for this tumor size group.

## Supplementary Information

Supplementary Figure S1.

Supplementary Figure S2.

Supplementary Table S1.

## Data Availability

Data available on request from the authors.
