# Supplementary material for: Microwave versus radiofrequency ablation for the treatment of liver malignancies: a randomized controlled phase 2 trial
Source: Sci Rep. 2022 Jan 10;12:316. doi: 10.1038/s41598-021-03802-x (PMC8748896; doi:10.1038/s41598-021-03802-x)
Supplement: Supplementary file 1 — Supplementary Figure S1. [file 41598_2021_3802_MOESM1_ESM.pdf]

Microwave versus radiofrequency ablation for the treatment of liver malignancies: a randomized controlled phase 2 trial

Aleksandar Radosevic[1*+], Rita Quesada[2+], Clara Serlavos[1], Juan Sánchez[1], Ander Zugazaga[1], Ana Sierra[1], Susana Coll[3], Marcos Busto[1], Guadalupe Aguilar[1], Daniel Flores[1], Javier Arce[1], José María Maiques[1], Montserrat García[3], José Antonio Carrion[3], Laura Visa[4], María Villamonte[5], Eva Pueyo[5], Enrique Berjano[6], Macarena Trujillo[7], Patricia Sánchez-Velázquez[5], Luís Grande[5], Fernando Burdio[5]

[+]These authors contributed equally to this work as co-first authors.

[1]Department of Radiology, Hospital del Mar, Barcelona, Spain; [2]Department of Experimental and Health Sciences, Universitat Pompeu Fabra, Barcelona, Spain; [3]Hepatology section, Gastroenterology Department, Hospital del Mar. IMIM. Barcelona, Spain.; [4]Department of Oncology-IMIM-Ciberonc, Hospital del Mar, Barcelona, Spain; [5]Department of Surgery, Hospital del Mar, Barcelona, Spain, [6]BioMIT, Department of Electronic Engineering, Universitat Politècnica de València, Valencia, Spain; [7]BioMIT, Department of Applied Mathematics, Universitat Politècnica de València, Valencia, Spain.

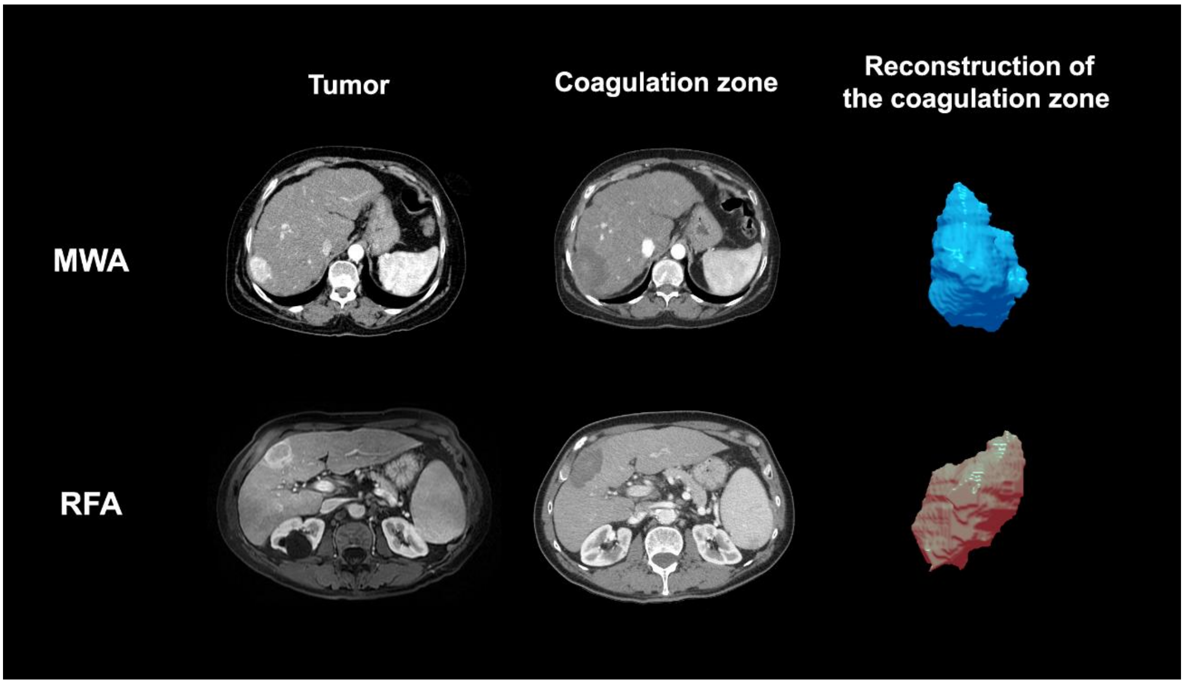

Figure S1 (Supplementary): Representative image of two HCC cases included in the trial with their pre- and post-treatment arterial phase axial images and 3D reconstruction of ablation zones (i.e. coagulation zones). Upper line (MWA Group): 4.0 × 2.2 × 3.2 cm (volume 19.8 cm^3) tumor with ablation zone of 6.5 × 4.0 × 6.1 cm (volume 71.4 cm^3). Bottom line (RFA Group): 3.8 × 2.4 × 3.3 cm (volume 14.1 cm^3) tumor with ablation zone of 6.1 × 2.9 × 5.1 cm (volume 37.6 cm^3).
