# Supplementary material for: Microwave versus radiofrequency ablation for the treatment of liver malignancies: a randomized controlled phase 2 trial
Source: Sci Rep. 2022 Jan 10;12:316. doi: 10.1038/s41598-021-03802-x (PMC8748896; doi:10.1038/s41598-021-03802-x)
Supplement: Supplementary file 2 — Supplementary Figure S2. [file 41598_2021_3802_MOESM2_ESM.pdf]

Microwave versus radiofrequency ablation for the treatment of liver malignancies: a randomized controlled phase 2 trial

Aleksandar Radosevic[1*+], Rita Quesada[2+], Clara Serlavos[1], Juan Sánchez[1], Ander Zugazaga[1], Ana Sierra[1], Susana Coll[3], Marcos Busto[1], Guadalupe Aguilar[1], Daniel Flores[1], Javier Arce[1], José María Maiques[1], Montserrat Garcia-Retortillo[3], José Antonio Carrion[3], Laura Visa[4], María Villamonte[5], Eva Pueyo[5], Enrique Berjano[6], Macarena Trujillo[7], Patricia Sánchez-Velázquez[5], Luís Grande[5], Fernando Burdio[5]

[+]These authors contributed equally to this work as co-first authors.

[1]*Department of Radiology, Hospital del Mar, Barcelona, Spain;* [2]*Department of Experimental and Health Sciences, Universitat Pompeu Fabra, Barcelona, Spain;* [3]*Hepatology section, Gastroenterology Department, Hospital del Mar. IMIM. Barcelona, Spain.;* [4]*Department of Oncology-IMIM-Ciberonc, Hospital del Mar, Barcelona, Spain;* [5]*Department of Surgery, Hospital del Mar, Barcelona, Spain,* [6]*BioMIT, Department of Electronic Engineering, Universitat Politècnica de València, Valencia, Spain;* [7]*BioMIT, Department of Applied Mathematics, Universitat Politècnica de València, Valencia, Spain.*

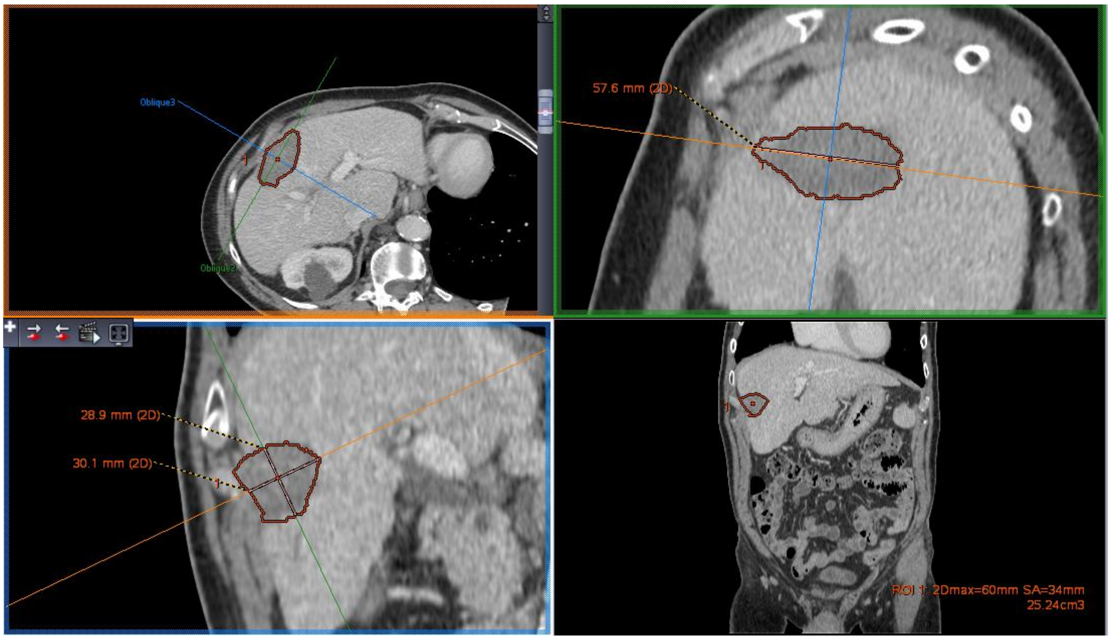

Figure S2 (Supplementary): Each ablation zone was semiautomatically segmented and the volume calculated automatically. The line of segmentation followed the border between the unenhanced ablated tissue and the surrounding, enhancing parenchyma. A 1-month portal phase CT scan images were used in all but two patients. For these two patients, the MRI images were used in a similar fashion. Three corresponding diameters were manually determined from multiplanar reconstruction following the applicator track.
