# Supplementary material for: Microwave versus radiofrequency ablation for the treatment of liver malignancies: a randomized controlled phase 2 trial
Source: Sci Rep. 2022 Jan 10;12:316. doi: 10.1038/s41598-021-03802-x (PMC8748896; doi:10.1038/s41598-021-03802-x)
Supplement: Supplementary file 3 — Supplementary Table S1. [file 41598_2021_3802_MOESM3_ESM.docx]

**Microwave versus radiofrequency ablation for the treatment of liver malignancies: a randomized controlled phase 2 trial**

Aleksandar Radosevic^1^*^+^, Rita Quesada^2+^, Clara Serlavos^1^, Juan Sánchez^1^, Ander Zugazaga^1^, Ana Sierra^1^, Susana Coll^3^, Marcos Busto^1^, Guadalupe Aguilar^1^, Daniel Flores^1^, Javier Arce^1^, José María Maiques^1^, Montserrat Garcia-Retortillo^3^, José Antonio Carrion^3^, Laura Visa^4^, María Villamonte^5^, Eva Pueyo^5^, Enrique Berjano^6^, Macarena Trujillo^7^, Patricia Sánchez-Velázquez^5^, Luís Grande^5^, Fernando Burdio^5^

^+^These authors contributed equally to this work as co-first authors.

*^1^Department of Radiology, Hospital del Mar, Barcelona, Spain; ^2^Department of Experimental and Health Sciences, Universitat Pompeu Fabra, Barcelona, Spain; ^3^Hepatology section, Gastroenterology Department, Hospital del Mar. IMIM. Barcelona, Spain.; ^4^Department of Oncology-IMIM-Ciberonc, Hospital del Mar, Barcelona, Spain; ^5^Department of Surgery, Hospital del Mar, Barcelona, Spain, ^6^BioMIT, Department of Electronic Engineering, Universitat Politècnica de València, Valencia, Spain; ^7^BioMIT, Department of Applied Mathematics, Universitat Politècnica de València, Valencia, Spain.*

**TABLE S1**. Unfavorable tumor location**

Data are n (number of tumors), n (%). *All data refer to tumors > 1.5 cm (see inclusion criteria). **See text for definition. Ablations

of local recurrences are not included. Analyses are by tumor. Depending on its location, the same tumor can be included into different subgroups.

|  | **MWA Group**  **(n=47)** | **RFA Group (n=50)** | **p-value** |
| --- | --- | --- | --- |
| Unfavorable location** | 34 (72%) | 37 (74%) | 0.854 |
| Subcapsular tumor | 27 (57%) | 28 (56 %) | 0.628 |
| Gall bladder | 3 (6%) | 7 (14 %) | 0.320 |
| Liver hilum | 7 (15%) | 3 (6%) | 0.191 |
| Gastrointestinal tract | 8 (17 %) | 6 (12%) | 0.482 |
| Diaphragm | 22 (46 %) | 19 (38%) | 0.331 |
| Pericardium | 5 (11 %) | 4 (8%) | 0.736 |
| Vessels > 3 mm | 12 (25 %) | 14 (28%) | 0.824 |
